# Structural insights into the functional diversity of the CDK–cyclin family

**DOI:** 10.1098/rsob.180112

**Published:** 2018-09-05

**Authors:** Daniel J. Wood, Jane A. Endicott

**Affiliations:** Newcastle Cancer Centre, Northern Institute for Cancer Research, Medical School, Newcastle University, Paul O'Gorman Building, Framlington Place, Newcastle upon Tyne NE2 4HH, UK

**Keywords:** cell cycle, kinase, cyclin, transcription

## Abstract

Since their characterization as conserved modules that regulate progression through the eukaryotic cell cycle, cyclin-dependent protein kinases (CDKs) in higher eukaryotic cells are now also emerging as significant regulators of transcription, metabolism and cell differentiation. The cyclins, though originally characterized as CDK partners, also have CDK-independent roles that include the regulation of DNA damage repair and transcriptional programmes that direct cell differentiation, apoptosis and metabolic flux. This review compares the structures of the members of the CDK and cyclin families determined by X-ray crystallography, and considers what mechanistic insights they provide to guide functional studies and distinguish CDK- and cyclin-specific activities. Aberrant CDK activity is a hallmark of a number of diseases, and structural studies can provide important insights to identify novel routes to therapy.

## 1. Introduction

Members of the cyclin-dependent protein kinase (CDK) family were originally characterized as serine/threonine-specific protein kinases activated by the expression of cyclin partners to drive the eukaryotic cell cycle [1]. Within the CMGC branch of the kinome, 20 proteins are now considered to be members of the CDK family that can be grouped into different phylogenetic sub-branches (see [2] for criteria for inclusion, illustrated and updated in [3]). In overview, in addition to those CDKs that regulate the cell cycle (CDKs 1, 2, 4 and 6), a substantial sub-branch of the family (CDKs 7, 8, 9, 12 and 13) regulates transcription through phosphorylation of the heptad repeats that comprise the C-terminal tail of RNA polymerase II (CTD) [4]. CDK7 is unusual in that it also indirectly regulates the cell cycle by activating CDKs 1, 2, 4 and 6 [5,6]. CDK3 phosphorylates retinoblastoma protein (pRB) to promote the transition from quiescence (G0) into G1 [7].

Other CDKs (CDKs 5, 10, 11, 14–18 and 20) have more diverse, CDK-unique functions that are frequently tissue-specific [8]. For example, CDK5 was one of the first CDKs to be characterized in non-cycling cells [9]. CDK10 is implicated in regulating gene transcription, but not through RNA pol II phosphorylation. It phosphorylates diverse substrates including the ETS2 oncoprotein and the protein kinase PKN2, and mutations in its cognate cyclin, cyclin M, result in STAR syndrome, a human developmental disorder [10,11]. CDK10 mutant and knockout mice also show growth and developmental delays [12]. CDK11–cyclin L complexes regulate RNA splicing, studied, for example, in the context of human immunodeficiency virus (HIV) transcript processing [13]. However, insights into these CDK–cyclin interactions are limited by the lack of structures for CDK10- and CDK11-containing complexes.

To partner the CDKs in humans, approximately 30 proteins are classified as cyclins [3,8]. The cyclins share very little sequence homology, but are structurally defined by the presence of either one or two copies of the cyclin box fold (CBF) [3,14]. The structures of monomeric CDK2 and cyclin A and of CDK2–cyclin A in various activation states were together taken to be a model for the regulation of the CDK family by cyclin binding and phosphorylation [15]. However, subsequent studies have shown that even closely related CDKs have distinct structural and sequence peculiarities. These differences translate into diverse substrate preferences and modes of regulation. CDK activity is wired into cell-type-specific signalling networks with the result that, taken together, knockout mice studies reveal both the redundancy inherent within the cell cycle CDKs, but also their tissue-specific activities ([16], CDK1; [17,18], CDK2; [19,20], CDK4; and [21,22], CDK6).

Dysregulation of CDK activity, either through activation of proteins that promote CDK activity or inactivation of oncogene-induced senescence pathways, is a common occurrence in various cancers [23–27]. Identifying and characterizing those cancers that require specific CDK activities for proliferation will provide the mechanistic understanding to better employ CDK-selective inhibitors. However, the importance of CDK activity to cancer initiation, growth and differentiation is further complicated by the emerging cell-cycle-independent roles of individual CDKs and cyclins in mammalian cells that are, respectively, cyclin and CDK partner-independent [28–30].

In this review, we compare and contrast the various monomeric CDK, CDK–cyclin and CDK-containing assemblies for which structures have been determined, and discuss how they might help to elucidate the different mechanisms that regulate CDK activity. Proteomic studies are identifying multiple proteins that bind to CDKs and cyclins that apparently do not share sequence features with proteins for which structures bound to CDKs or cyclins are available (table 1). A comparison of the structures of CDK–cyclin complexes reveals how the CDK and cyclin partners can differ in their relative disposition and the alternative surfaces that can be exploited to recognize CDK substrates and regulators. The extent to which protein interaction sites are conserved and recycled within the CDK and cyclin families is yet to be fully explored, but will be reviewed here. The kinetic and catalytic mechanism of protein kinases including CDK2 was reviewed in 2012 [31]. The structures of CDK–cyclin complexes bound to ATP-competitive inhibitors have also been reviewed recently [32], and these will only be discussed in so far as they give insights into functionally significant conformations.

**Table 1.** CDK-containing complexes deosited in the Protein Data Bank (PDB).

CDK	partners^a^
CDK1	cyclin B, Cks1, Cks2
CDK2	cyclin A/B/E, KAP, Cks1, p27KIP1, Spy-1
CDK4/6	cyclin D (structurally CDK4–cyclin D), viral cyclin (CDK6), p16INK4A (CDK6), p19INK4D (CDK6), HSP90–Cdc37 (CDK4), p18INK4C–cyclin K (CDK6)
CDK5	p25
CDK8	cyclin C
CDK9	cyclin T, Tat, AFF4, TAR
CDK12	cyclin K
CDK13	cyclin K

^a^Partner proteins included in the table are those for which CDK-complex structures have been deposited in the Protein Data Bank.

## 2. Relating structure and function

### 2.1. The inactive monomeric CDK fold

**Figure 1.**
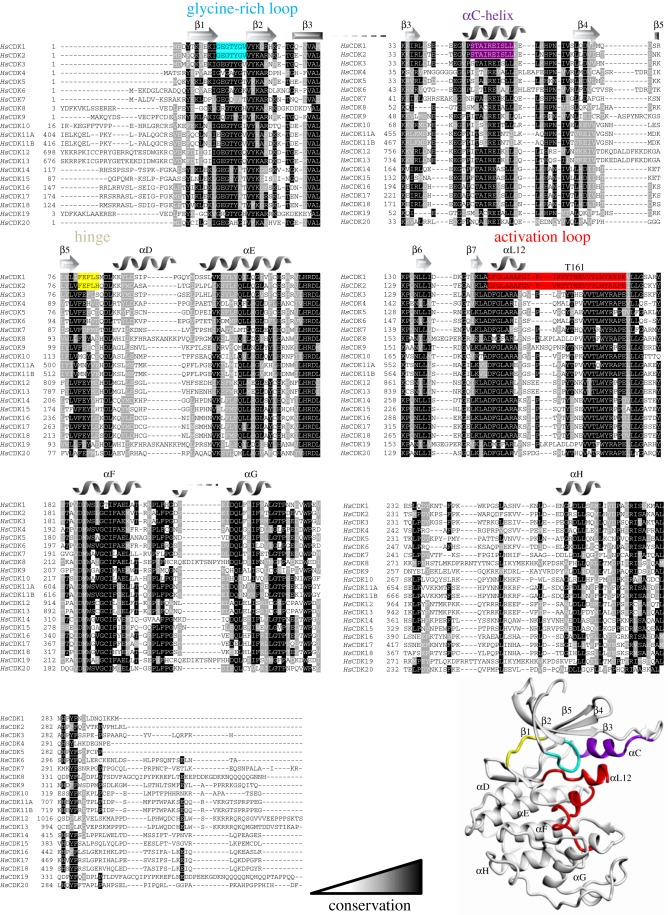
Sequence alignment of the human CDK family. Greyscale shading denotes the extent of sequence conservation calculated from UniProt sequences using Clustal Omega [33] and exported into ExPASy BoxShade. Structural features described in the text are named and highlighted in colour above the alignment and located on the fold of CDK1 (extracted from the CDK1–Cks1 complex, PDB code: 4YC6). UniProt codes used: CDK1 (P06493), CDK2 (P24941), CDK3 (Q00526), CDK4 (P11802), CDK5 (Q00535), CDK6 (Q00534), CDK7 (P50613), CDK8 (P49336), CDK9 (P50750), CDK10 (Q15131), CDK11A (Q9UQ88), CDK11B (P21127), CDK12 (Q9NYV4), CDK13 (Q14004), CDK14 (O94921), CDK15 (Q96Q40), CDK16 (Q00536), CDK17 (Q00537), CDK18 (Q07002), CDK19 (Q9BWU1), CDK20 (Q8IZL9). CDK11A and CDK11B result from a gene duplication and are almost identical (97.5%).

**Figure 2.**
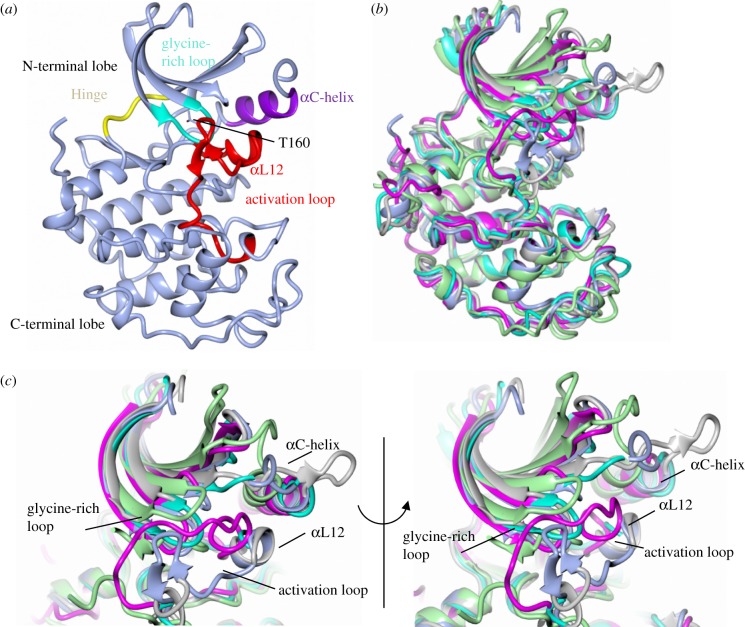
The monomeric CDK fold. (*a*) Structure of monomeric CDK2. The CDK kinase fold, as first exemplified by monomeric CDK2 ([39], PDB 1HCK), is composed of a smaller N-terminal lobe that is predominantly a twisted anti-parallel β-sheet linked via a flexible hinge sequence to a larger C-terminal lobe dominated in structure by α-helices (light blue ribbon). Structural features are highlighted: glycine-rich loop (sequence GXGXXG, cyan), αC-helix (residues 45–55, purple), hinge (residues 80–84, yellow), activation loop (residues 145–172, red). The location of T160 is marked. (*b*) The monomeric CDK fold is conserved as shown by an overlay of CDK1 (extracted from the structure of CDK1–Cks2), CDK2, CDK6, CDK7 and CDK16 structures. The other CDK folds are superposed on CDK2: CDK1 (PDB 4YC6, light grey); CDK6 (PDB 5L2S, cyan); CDK7 (PDB 1UA2, magenta) and CDK16 (PDB 5G6 V, light green). Mobility is indicated by the quality of the experimental electron density maps, so that the derived structures can be traced with varying degrees of confidence. (*c*) The various conformations the activation and glycine-rich loops can adopt are highlighted by this structural comparison. Structures reported for these loops may represent more populous low energy conformations compatible with a particular crystal lattice. This model is supported by studies of monomeric CDK2 phosphorylated on the conserved threonine residue within the activation loop (T160 in CDK2), which exhibits approximately 0.3% of the fully active CDK2–cyclin A complex ([40], PDB 1QMZ). The majority of the CDK2 probably corresponds to inactive conformations, but a small fraction is in an active conformation and generates the basal activity observed.

CDKs vary in the lengths of N- and C-terminal sequences that bookend the conserved, central protein kinase domain [8] (figure 1). Overall, the structures of cyclin-free CDK1 ([34], PDB 4YC6), CDK2 ([35], PDB 1HCK), CDK6 ([36], e.g. PDB 5L2S), CDK7 ([37], PDB 1UA2) and CDK16 ([38], PDB 5G6 V) superimpose very well. For example, monomeric CDK2 and CDK7 overlay with an r.m.s.d. (root-mean-square deviation) of 1.49 Å over 262 equivalent Cα atoms. They share conserved structural features that ensure they are catalytically inactive (figure 2*a*). The start of the activation loop (defined as the sequence between the conserved DFG and APE motifs, residues 145–172 in CDK2) adopts a short α-helical conformation (αL12) that blocks the C-helix from swinging in to reshape the back of the active-site cleft. A characteristic of the glycine-rich (residues 12–16 in CDK2 that encodes the conserved GXGXXG motif) and activation loops is their relative mobility. As a result, differences between cyclin-free CDK structures are most evident around the active site (figure 2*b*,*c*). Accompanying these changes are more subtle differences in the relative dispositions of the N- and C-terminal lobes that lead to other conserved residues within the catalytic sites adopting positions that are incompatible with catalysis (figure 2*b*).

The classical model of CDK activation exemplified by CDK2–cyclin A is not applicable to the CDK5-related sub-branch of the CDK family of which CDK16 is a member [3]. There are several emerging unusual features of CDK16 activation that would benefit from structural characterization. A CDK16 feature that it shares with CDKs 14, 15, 17 and 18 is an extended N-terminal regulatory region before the start of the kinase domain. This sequence is important for CDK16 association with its cognate cyclin, cyclin Y or cyclin Y-like 1 [3,41–43]. In addition, stable association of cyclin Y with either CDK14 [44] or CDK16 [45] requires cyclin Y phosphorylation and binding to 14-3-3, suggesting that a classical bidentate 14-3-3–ligand interaction [46] may help to organize cyclin Y to bind to its cognate CDK partner.

### 2.2. CDK2–cyclin A activation

**Figure 3.**
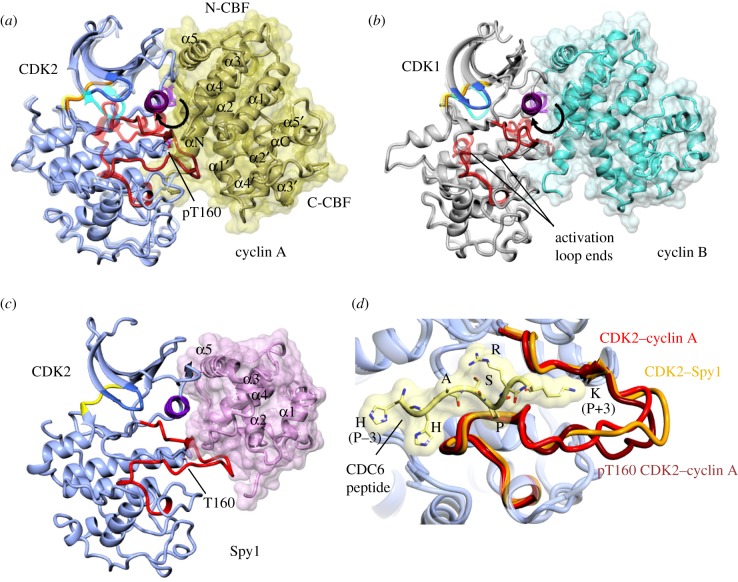
CDK activation by cyclin binding. (*a*) Overlay of monomeric CDK2 and T160-phosphorylated CDK2–cyclin A. Cyclin A composed of two tandem cyclin box folds (CBFs [49], PDB 1VIN) acts as a scaffold to which the malleable unphosphorylated CDK responds to generate a binary complex that exhibits basal activity ([47], PDB 1JST). The CDK αC-helix is rotated and relocated into the active site by engagement with the N-CBF of the cyclin subunit. At the start of the activation loop, αL12 is melted and the conserved DFG motif adopts an active ‘DFG-in’ conformation in which the aspartate side chain coordinates a magnesium ion to productively orientate the ATP phosphate groups for catalysis. The activation loop is extended and pulled away from the active site to form a platform that will ultimately recognize the protein substrate around the site of phospho-transfer ([50], PDB 1QMZ). Cyclin binding also refines the relative positions of the CDK2 N- and C-terminal lobes, so that residues within the hinge and lining the active site orientate the ATP adenine and ribose rings and phosphate groups for catalysis. Overall, the CDK2–cyclin A interface is extensive (2839 Å^2^, [51]) extending between both lobes of the CDK and the two cyclin CBFs, further strengthened by engagement of the cyclin N-terminal helix preceding the N-CBF with the CDK C-terminal lobe. The phospho-threonine within the activation loop (T160 in CDK2) acts as a structural hub liganded by conserved, positively charged residues located within the C-helix (R50), at the start of the activation loop (R150) and adjacent to the catalytic aspartate residue (R126). In the absence of T160 phosphorylation, a conserved C-terminal glutamate residue (E162 in CDK2) satisfies the positively charged side chains of the phospho-threonine-binding pocket, and the side chain hydroxyl of T160 is solvent accessible within the context of a relatively well-ordered activation loop ([47], PDB 1JST). The inactive conformation of CDK2 is shown as a translucent ribbon. The N-CBF and C-CBF are also shown. (*b*) CDK1–cyclin B (PDB 4YC3; CDK1 grey, cyclin B translucent cyan surface). Inactive (cyclin-unassociated) CDK1 conformation shown as a translucent ribbon. (*c*) CDK2–Spy1 is shown in a similar pose (PDB 5UQ2; CDK2 blue, Spy1 translucent pink surface). (*d*) Comparison of unphosphorylated CDK2–cyclin A (PDB 1FIN; activation loop, red), T160-phosphorylated CDK2–cyclin A with peptide present (PDB 2CCI; peptide, yellow activation loop, deep red) and CDK2–Spy1 (PDB 5UQ2; activation loop in brown) activation loop conformations. The positions of residues (P−3 to P+3) within the CDC6 peptide substrate (sequence HHASPRK) with respect to the serine residue at the site of phospho-transfer (P position) are indicated.

CDK2 partners cyclin E during late G1 and is subsequently bound to cyclin A during S-phase for DNA replication [1]. A series of structures of CDK2 bound to cyclin A provided snapshots of the structural changes that accompany cyclin binding and phosphorylation of the CDK2 activation loop [39,47,48] (figure 3*a*). Subsequent studies that have interrogated the kinetics of CDK2 activation in a cellular context have demonstrated that CDK-activating kinase (CAK, a complex of CDK7 and cyclin H in humans) is active against CDK2 (i.e. through phosphorylation of CDK2 T160), which is then proposed to bind to cyclin A [52]. This result suggests a model in which flexibility around T160 is required for CDK2 to be recognized by CAK and that the adoption of an ordered activation loop conformation accompanies anchoring of the phospho-threonine residue promoted by cyclin binding.

The flexibility of the CDK fold has also been captured in ATP-competitive inhibitor-bound structures where inhibitor binding helps to stabilize alternative energetically less favourable conformations. At the start of the activation loop, the conserved DFG motif can adopt either an active ‘DFG-in’ conformation (figure 3), or an inactive ‘DFG-out’ conformation in which the phenylalanine side chain points into the active-site cleft and is removed from its position in the ‘regulatory spine’ of residues that characterizes the active protein kinase fold [53]. This latter conformation has been exploited for the design of several tyrosine kinase-specific inhibitors [54,55]. Though the majority of CDK ATP-competitive inhibitor structures determined to date have a ‘DFG-in’ conformation [32], inhibitor binding to monomeric CDK2 ([56], PDB 5A14) and monomeric CDK16 ([38], PDB 5G6 V) and to cyclin-bound CDK8 (PDB 3RGF) can stabilize the CDK fold into a ‘DFG-out’ conformation. Thus, the binding of ATP-competitive inhibitors interrogated by the determination of multiple ‘snapshots’ of protein kinase structures highlights the inherent flexibility of the CDK fold and its ability to adopt multiple conformations [31,55].

### 2.3. Extending the activation model to other cyclin partners of CDK1 and CDK2

CDK1 is the closest member of the CDK family to CDK2 and for which structures of the cyclin-free and authentic cyclin-bound forms can also be compared (figure 3*b*; [34], PDB codes 4YC6 and 4YC3). It is the only essential CDK and, activated by its partners cyclins A and B, it executes progression through mitosis. Overall, the mechanism of CDK1 activation is conserved with CDK2. However, an opening of the interface coupled with a twist between the two proteins relative to CDK2–cyclin A results in a re-orientation of the C-helix and fewer interactions between the cyclin B and CDK1 C-terminal lobes. Overall, the interfacial surface is 30% smaller in CDK1–cyclin B compared with CDK2–cyclin A. Crystallographic electron density maps of unphosphorylated CDK1 suggest that it has a more flexible activation segment than does the comparable state of CDK2.

A comparative analysis of the sequence loci that mediate the CDK1– and CDK2–cyclin interfaces reveals the conserved sequence features that may explain CDK1 and CDK2 cyclin selectivity [34]. CDK2 is partnered by cyclin E during late G1 phase and then subsequently by cyclin A [1]. Under circumstances where CDK1 expression is knocked down, it can also partner cyclin B [57]. A comparison of the structures of phosphorylated CDK2 bound to cyclin A ([48], PDB 1JST), cyclin B [58], PDB 2JGZ) and cyclin E ([51], PDB 1W98) revealed the conserved nature of the CDK2 response to cyclin binding [34]. Cyclins A and B conserve three large aromatic residues at the CDK–cyclin interface (Y170, Y177 and Y258 in cyclin B), whereas in cyclin E the residues at these positions have smaller side chains (N112, I119 and L202). Given the smaller CDK1–cyclin interface compared with CDK2–cyclin A, the structures would predict that CDK1 would bind preferentially to cyclins B and A, but that these smaller side chains would have less impact on CDK–cyclin affinity in the context of the larger CDK2–cyclin interface.

A comparison of the CDK1–cyclin B and CDK2–cyclin A/B/E structures also highlights the potential for these closely related CDKs to be differentially regulated by reversible phosphorylation. The antagonistic activities of Wee1/Myt1 kinases and Cdc25 phosphatases regulate the phosphorylation status of the CDK glycine-rich loop (defined by the GXGXXG motif, residues 11–16 in CDK2). The structure of CDK2–cyclin A phosphorylated on Y15 illustrates how phosphorylation promotes a glycine loop structure that antagonizes both peptide substrate binding and the ATP conformation required for catalysis [59]. The flexibility of the glycine-rich loop is compatible with a model in which the phosphorylated Y15 side chain is solvent exposed and accessible to both kinases and phosphatases. CDK1 is also regulated by active-site phosphorylation, and the conserved nature of the structure in this region suggests that the mechanism of inhibition is also conserved.

However, unlike the glycine-rich loop, the flexibilities of the phosphorylated CDK1 and CDK2 activation loops differ. Though the structure of a T161-phosphorylated CDK1–cyclin B complex is yet to be determined, this complex is susceptible to phosphatase treatment, suggesting that the phosphorylated CDK1 activation segment remains flexible [34]. By contrast, phosphorylated CDK2 T160 is embedded within a network of ionic interactions (figure 3) that orders the CDK2 activation segment within this region and decreases T160 solvent accessibility. Taking Y15 as the model, this difference could ensure that the activity of CDK1, more so than that of CDK2, remains subject to the ongoing antagonistic activities of CAK and phosphatases. In particular, it would offer an opportunity for CDK1 to be subject to rapid enzyme-mediated inactivation even in the presence of high concentrations of cyclin B and might offer a regulatory opportunity to distinguish CDK1 and CDK2 activities.

Ringo/Spy proteins also activate CDK1 and CDK2 and represent a divergent branch of the cyclin family, identified through their ability to induce meiotic maturation in *Xenopus* oocytes [60,61], an activity conserved in humans [62]. Ringo A/Spy1 is required for localizing CDK2 to telomeres, and its absence results in defects in chromosome tethering to the nuclear envelope [63,64]. Several studies have implicated Spy1 in glioma, suggesting that it may also have functions in mitosis in selected cell types [65]. Ringo A knockout mice show similar defects to CDK2 knockout mice during spermatogenesis [63], suggesting that the essential function of CDK2 during meiosis might be mediated, in part, by its association with Ringo A. Spy1 (Ringo A) encodes only a single CBF embedded within a longer sequence and activates CDK2 through a mechanism that does not require activation loop phosphorylation (figure 3*c*; [66], PDB 5UQ2). Immediately after the DFG motif, CDK2 R157 and T158 anchor the activation loop through electrostatic interactions with Spy1 D97 and E135, respectively. CDK2 R50 and R150 that coordinate the phosphorylated CDK2 T160 side chain in the CDK2–cyclin A structure interact with Spy1 D136, so that its carboxylate moiety effectively mimics a number of interactions made by the phosphoT160 phosphate group. These alternative interface interactions create a CDK2 activation loop conformation most reminiscent of that seen when it is bound to cyclin A (figure 3*d*). The resulting complex has measurable kinase activity but is less active than phosphorylated CDK2–cyclin A [66].

### 2.4. Comparison of the crystal structures of CDK–cyclin complexes

To what extent the mechanism for CDK activation proposed through studies on CDK2 can be extended to other members of the CDK and cyclin families has been challenged by further CDK–cyclin structures. Cyclin-free structures are not available for other CDKs determined in their cognate cyclin-bound states, so inferences about the mechanism of activation can only be made by presuming a conserved inactive monomeric CDK fold. Taken together, they provide diverse examples of how CDK activation can be achieved; models for activation of CDK5 and CDK4, in particular, are quite distinct.

#### 2.4.1. CDK4 and CDK6

**Figure 4.**
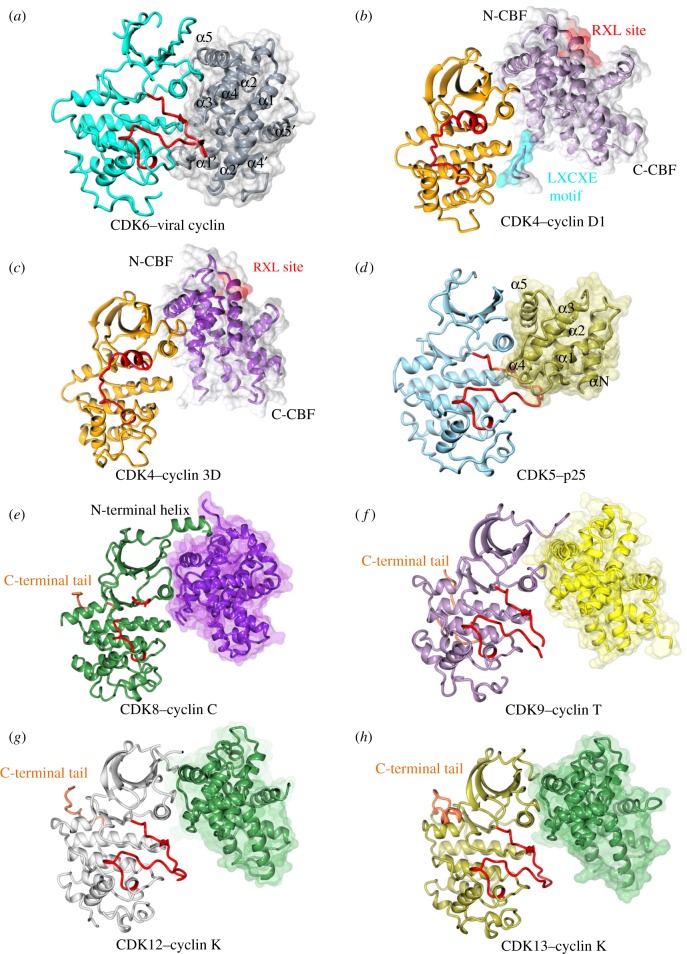
CDK–cyclin complexes. A comparison of the CDK–cyclin complexes, for which structures are available, highlights the differences in the CDK response to cyclin association. (*a*) CDK6–viral cyclin (PDB 1JOW, CDK6, cyan with activation loop (residues 163–189) shown in red; viral cyclin, grey). (*b*) CDK4–cyclin D1 (PDB 2W96, CDK4, orange; cyclin D1, light purple, RXL-binding site shown as a red translucent surface (residues 54–61) and partially resolved LXCXE motif shown in cyan (residues 6–9)). (*c*) CDK4–cyclin D3 (PDB 3G33, CDK4, orange; cyclin D3, purple, RXL-binding site shown as a red translucent surface (residues 56–61). (*d*) CDK5–p25 (PDB 1H4 L, CDK5, light blue with activation loop (residues 144–171) shown in red; p25, gold). (*e*) CDK8–cyclin C (CDK8, green with C-terminal residues 343–353 in orange; cyclin C, purple). (*f*) CDK9–cyclin T1 (PDB 3BLH, CDK9 lilac with C-terminal residues 317–325 in orange; cyclin T, pale yellow). (*g*) CDK12–cyclin K (PDB 4UN0, CDK12, light grey, C-terminal rail residues 1025–1036 in orange; cyclin K, green). (*h*) CDK13–cyclin K (PDB 5EFQ, CDK13, gold, C-terminal tail residues 1011–1025 in orange; cyclin K, green). The activation segment sequences are shown in red where resolved in the structures.

CDK4 and CDK6 are frequently considered together as promoters of G1 progression. In this context, they phosphorylate relatively few substrates, notably the retinoblastoma protein, its relatives and a number of transcription factors [67]. A structure for a CDK6–cyclin D complex has not been determined, but CDK6 bound by a viral cyclin provides another illustration of how an alternative CDK–cyclin interface generates an active CDK conformation in the absence of activation segment phosphorylation (figure 4*a*; [68], PDB 1JOW). Viral cyclin binding re-organizes the CDK6 C-helix and ensures that the path of the activation segment C-terminal to T177 (equivalent to CDK2 T160) forms a peptide-binding platform equivalent to that seen in CDK2. A novel β-sheet interaction made between the CDK6 sequence preceding T177 and the viral cyclin N-terminal sequence, that has no counterpart in any other known CDK–cyclin complex structure, stabilizes the activation segment.

The structures of non-phosphorylated and phosphorylated CDK4 bound to cyclin D3 ([69], PDB 3G33; figure 4*c*) or cyclin D1 ([70], PDB 2W96; figure 4*b*), respectively, revealed that the structural mechanism of CDK4 activation must be distinct from that of CDK1 or CDK2. Only the cyclin D N-terminal CBF (N-CBF) binds to CDK4, the C-terminal lobes of both proteins are splayed apart to create a solvent-filled cleft between the two subunits. Cyclin D binding does not induce an active CDK4 conformation. In both structures, the CDK4 C-helix remains displaced, reminiscent of cyclin-free CDK1 and CDK2, and the activation loop is either largely disordered (CDK4–cyclin D3) or adopts a conformation that occludes the active site and is incompatible with substrate binding (CDK4–cyclin D1). Based on these structural insights, a substrate-assisted model of CDK4–cyclin D catalysis has been proposed in which substrate engagement with the cyclin at the RXL site (see below) promotes the transient folding of the CDK4 into an active conformation [69].

The CDK4 activation loop remains accessible to cycles of phosphorylation and dephosphorylation by CAK and phosphatases, respectively [69]. In cells, sustained CAK activity is required to maintain CDK4 and CDK6 activity [71], an observation supported by the CDK4–cyclin D structures. It can be hypothesized that CDK6 bound to cyclin D1, D2 or D3, in contrast to the structure it adopts bound to a viral cyclin (described above), might also retain flexibility in the activation loop around T177. Whether CDK6–cyclin D resembles CDK4–cyclin D1/D3 or alternatively accommodates more local activation loop flexibility in the context of a cyclin-activated structure (i.e. more reminiscent of the structure of CDK1–cyclin B) will require the determination of the structure of CDK6 bound to a cognate cyclin. The conserved nature of the CDK4/6 active sites and their ability to adopt similar structures is exemplified by the successful recent registration for clinical use of highly selective ATP-competitive CDK4/6 inhibitors [72].

However, there are structural differences between CDK4 and CDK6 that can impact function. For example, whereas CDK6 is a relatively weak client of the Hsp90–Cdc37 pathway, CDK4 is a strong client [73–76] and many of its partner proteins regulate protein folding and complex assembly [77]. These differences in stability are reflected in the affinities of CDK4 and CDK6 for various regulatory proteins [78]. Taken together, these results suggest that CDK4 is an unstable protein that is prone to unfolding and whose integrity is dependent on protein association, a model further substantiated by structural studies of a CDK4–Cdc37–Hsp90 complex [79] (see below).

#### 2.4.2. CDK5

CDK5 is expressed in post-mitotic neuronal cells where it binds to p35 and p39 and phosphorylates key regulators such as tau and β-APP [9]. Dysregulation of CDK5 activity was initially characterized in the context of neurodegenerative diseases and neurological disorders [80], although there is increasing evidence that, in certain cellular contexts, it can also contribute to tumorigenesis [81,82]. p35 proteolysis promoted by neurotoxic conditions generates p25, a C-terminal fragment that retains the ability to activate CDK5. p25 encodes eight α-helices that have a related but distinct topology when compared with the cyclin A N-CBF (figure 4*d*; [83], PDB 1H4 L). Overall, given their different relative helical dispositions, it is difficult to make direct comparisons between the cyclin A and p25-mediated CDK interfaces, except that they both stabilize an active CDK conformation. Two loops linking p25 α1 to α2 and α3 to α4 make extensive contacts with the CDK5 activation segment and stabilize a non-phosphorylated active conformation. Within this region, CDK5 has three arginine residues spatially equivalent to the three arginines that coordinate CDK2 phosphoT160, and two of them (R50 and R149) are alternatively employed at the p25 interface.

CDK5 can also bind to cyclin E [84]. The adult brain expresses high levels of cyclin E, which can compete with p35 for CDK5 and inhibit CDK5 activity. In its absence, unrestrained CDK5–p35 activity can lead to pathological synapse growth, and formation of CDK5–cyclin E complexes promotes synapse formation. A number of CDKs have multiple authentic cyclin partners that post-CDK activation can impose distinct substrate preferences on their CDK partner. However, this example is distinguished in that cyclin binding inhibits CDK activity.

#### 2.4.3. Transcriptional CDKs, CDK8, CDK9, CDK12 and CDK13

Within the transcriptional CDKs sub-branch, CDKs 7, 8/19 and 9 are found, respectively, as components of TFIIH, the mediator complex CDK8 kinase module (or its paralogous complex containing CDK19) and positive transcription elongation factor b (P-TEFb). Collectively, they phosphorylate both specific residues within the heptad repeats that constitute the CTD (CDKs 7 and 9) and associated factors (CDKs 7, 8/19 and 9). CDK7 [85] and CDK8 [86,87] regulate the initiation of transcription and CDK9 subsequent release from promoter proximal arrest [88] (reviewed in [89]). CDK12 [90–92] and CDK13 [93] bound to cyclin K are associated with transcript synthesis towards the middle and 3′-end of the emerging RNA, at which point they phosphorylate the CTD-heptad repeats. CDK12–cyclin K also regulates alternative last exon splicing [94].

CDK12–cyclin K promotes pre-replicative complex formation during G1 by regulating the activity of cyclin E1 [95]. CDK12–cyclin K has also been reported to regulate the expression of a subset of genes that mediate the DNA damage response [91] and CDK13 gene sets that are involved in growth signalling [93]. Mutations in CDK13 are associated with developmental heart defects and intellectual development, suggesting it is required for the execution of specific gene expression programmes [96]. To what extent these CDKs balance activities as part of the core machinery of RNA pol II-dependent transcript processing against activity on subsets of genes is yet to be fully characterized. A characteristic of CDKs 12 and 13 is the presence of much longer sequences N- and C-termini to the conserved catalytic fold than is found in other transcriptional CDKs (figure 1). These sequences are as yet not structurally characterized but do contain a number of arginine/serine-rich and proline-rich motifs (amongst others) and regulate CTD phosphorylation [97].

CDK–cyclin structures have been determined for a substantial subset of the transcriptional branch of the CDK family, CDK8 bound to cyclin C ([98], PDB 4F7S; figure 4*e*), CDK9 bound to cyclin T ([99], PDB 3BLH; figure 4*f*) and CDK12 ([100], PDB 4UN0; figure 4*g*) and CDK13 ([93], PDB 5EFQ; figure 4*h*) bound to cyclin K. CDK8, CDK9 and CDK12 are reminiscent of CDK4 and engage their cyclin partners almost exclusively through their respective CDK and cyclin N-terminal lobes. However, the CDK8–cyclin C interface is made more substantial by additional interactions between an N-terminal helix present in CDK8 that recognizes the cyclin C N-CBF. The CDK12–cyclin K interface is also more extensive than that between CDK9 and cyclin T, mediated by further interactions between the CDK12 N-terminal lobe and the N-terminal region of cyclin K. Cyclin T binding and activation loop phosphorylation creates a CDK9 peptide-binding platform reminiscent of that seen in CDK2–cyclin A [99]. Interestingly, these three cyclin-bound CDKs differ in their activation mechanisms: CDK9 can autophosphorylate *in cis* on T186 *in vitro* [99], but *in vivo* phosphorylation is CDK7-dependent [101], as is phosphorylation of CDK12 [102]. CDK8 is active in the absence of activation loop phosphorylation [103].

A more detailed structural comparison highlights other structural differences that impact activity and regulation. The first CDK8–cyclin C structure (PDB 3RGF) was crystallised in the presence of sorafenib which imposed a ‘DMG-out’ conformation at the start of the CDK8 activation loop [103]. A substantial fraction of the following activation loop sequence proved to be flexible and could not be built between residues R178 and V195, encompassing the predicted peptide substrate-binding site. Subsequent structures of apo CDK8–cyclin C (PDB 4F7S, [98]) and other CDK8–cyclin C–ATP-competitive inhibitor structures in a ‘DMG-in’ conformation ([98,104], PDB 4CRL; [105], PDB 5CEI) were also disordered in this activation loop region. Notably, the CDK8-specific loop linking helices αF and αG (residues 239–247), which lies below the activation loop, is also disordered. These observations suggest that association with other components of the Mediator complex may be required to stabilize the CDK8 structure in this region to activate its activity.

**Figure 5.**
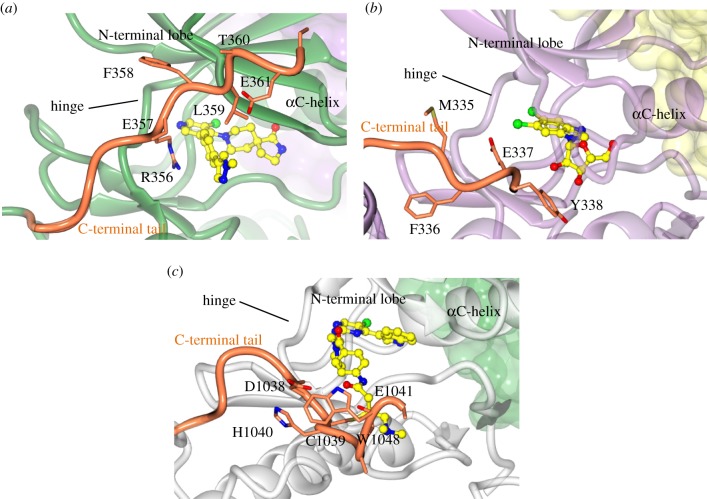
Shaping of the catalytic cleft by the C-terminal tail in the transcriptional CDKs. In each case, the binding of an ATP-competitive inhibitor (ball and stick model) within the ATP-binding pocket helps to order the C-terminal sequence. (*a*) CDK8–cyclin C–CCT251545 (PDB entry 5BNJ); (*b*) CDK9–cyclin T1–DRB (PDB 3MY1) overlaid with the full-length CKD9 structure (PDB 4RC8); (*c*) CDK12–cyclin K–THZ531 (PDB 5ACB). In this structure, the inhibitor THZ531 forms an irreversible bond with C1039 located within the CDK12 C-terminal extension. The CDK8, 9 and 12 folds are coloured green, lilac and grey, respectively, and the CDK C-terminal tails are coloured orange. The hinge region between the N- and C-terminal kinase lobes and the αC-helix is identified to provide the context.

Taken together, the transcriptional CDKs are all characterized by having an extended, flexible C-terminal tail beyond the kinase catalytic core fold (figure 4*e–h*). Where structures have been determined, they reveal that this sequence impacts the character of the ATP-binding site (figure 5). The CDK9 C-terminal tail is anchored by conserved residues F336 and E337 that bind, respectively, into a hydrophobic pocket just before the hinge sequence and into the ATP-binding site ([106], PDB 4EC8). A model can be proposed that, during the catalytic cycle, the active, closed-state conformation is stabilized by folding of the C-terminal tail, generating a fully enclosed active site bounded on one side by the C-terminal tail and on the other by the peptide substrate. Notably, CDK9 follows an ordered reaction mechanism in which ATP binds first and ADP is released last [106]. Mutation of F336 and E337 to alanine or deletion of the C-terminal tail converts the mechanism to a random one (cf. CDK2 or CDK5, [107]), suggesting that conformational cycling of the tail sequence imposes reaction order. This kinetic analysis supports a distributive rather than processive mechanism for CTD phosphorylation by P-TEFb (see also [108]), which might impact the distribution of phosphorylation events on the CTD sequence [109,110]. Substrate (ATP) trapping in a closed state is a feature of a CDK12–cyclin K-AMP–PNP complex (PDBs 4NST [102]; and 4CXA [100]) and of a CDK13–cyclin K–ATP complex where residues within the tail make direct interactions with ATP ([93], PDB 5EFQ). The binding of various ATP-competitive inhibitors also orders the CDK8 C-terminal tail (figure 5*a*) ([104], PDB 4CRL; [105], PDB 5CEI; [111], PDB 5IDN; [112], PDB 5BNJ; and [113], PDB 5HVY). Beyond its ability to shape the ATP-binding site, it remains to be determined to what extent the conformational flexibility of the C-terminal tail is employed as a structural mechanism to regulate this sub-branch of the CDK family.

## 3. CDK substrate recognition

The structure of CDK2–cyclin A bound to a non-hydrolysable ATP analogue and an optimal substrate peptide (HHASPRK) revealed how the activation segment is modelled to recognize a proline residue at the P + 1 position and a positively charged residue at P + 3 (where P is the phosphate-accepting residue) ([50], PDB 2CCI; figure 3*d*). Structural studies support a dissociative mechanism through a metaphosphate intermediate in which the attacking group (serine or threonine hydroxyl) from the peptide substrate comes in opposite to the leaving group (phosphate ester oxygen of the γ-phosphate group of ATP), leading to inversion of configuration at the phosphorus (PDB codes: 3QHR and 3QHW [114], and 1GY3 [115]). Apart from this motif, the only other significant sequence feature shared by many cell cycle CDK substrates is the RXL motif, first identified by comparative sequence analysis of multiple CDK substrates and inhibitors [116]. This sequence binds to a site on the cyclin N-CBF that is conserved between cyclins A, B, D and E, and was first structurally characterized following the determination of the structure of a CDK2–cyclin A–p27KIP1 complex (PDB 1JSU, [117]).

A feature of the cyclin B-bound CDK1 is the retention of flexibility within the activation loop upon T161 phosphorylation [34] (figure 3*b*). Using a series of model peptide substrates, a comparative activity study suggested that for CDK1, this enhanced flexibility translates into a more relaxed substrate preference around the site of phospho-transfer [34]. In the presence of an RXL motif, CDK1 will phosphorylate motifs that contain either a proline residue at P + 2 or a positively charged residue at P + 3. CDK1 is characterized by its promiscuous ability to phosphorylate a wide variety of substrates at multiple sites, many of which are ‘non-canonical’ [116,118–120]. The structure of CDK1 suggests a mechanism by which activation loop flexibility, embedded in an inherently, more flexible CDK1 fold allows CDK1 to accommodate a more diverse substrate set than its nearest relative CDK2. These plastic properties may also contribute to its ability to partner non-cognate cyclins in the absence of other CDKs to drive the cell cycle [34,121].

The structures of CDK4 bound to cyclin D1 and cyclin D3 support a model in which a catalytically competent active-site configuration must occur transiently when CDK4–cyclin D forms a Michaelis complex with ATP and protein substrates (figure 4*b*,*c*). Purified CDK4–cyclin D3 requires the presence of an RXL motif within the peptide substrate for activity, suggesting that substrate engagement through the cyclin recruitment site promotes both productive substrate engagement and kinase remodelling. Such a substrate-assisted catalysis model would be supported by kinetic studies in which CDK4 has been shown to follow an ordered sequential mechanism in which ATP binds first and the phospho-peptide product leaves last [122]. CDK4/6–cyclin D complexes monophosphorylate pRB at multiple sites and further hyperphosphorylation is mediated by CDK2–cyclin E [123]. Although it is not clear what function monophosphorylation performs, taken together, these observations suggest that CDK4 activity is more tightly regulated by substrate scaffolding than CDK1 and CDK2. Whether the model extends to CDK6 awaits the determination of the structure of CDK6 bound to an authentic D-type cyclin.

The RXL-binding cyclin recruitment site was the first to highlight the use of substrate docking sites to enhance CDK activity towards particular substrates [124–126]. Permutations on this sequence can be accommodated with differing affinities by cyclins to refine substrate recognition [58,127,128]. Compatible with a docking model, crystallographic attempts to determine a substrate path between the RXL and SPXK motifs for the binding of a model substrate to CDK2–cyclin A failed to resolve electron density for residues beyond the consensus sequences [129].

**Figure 6.**
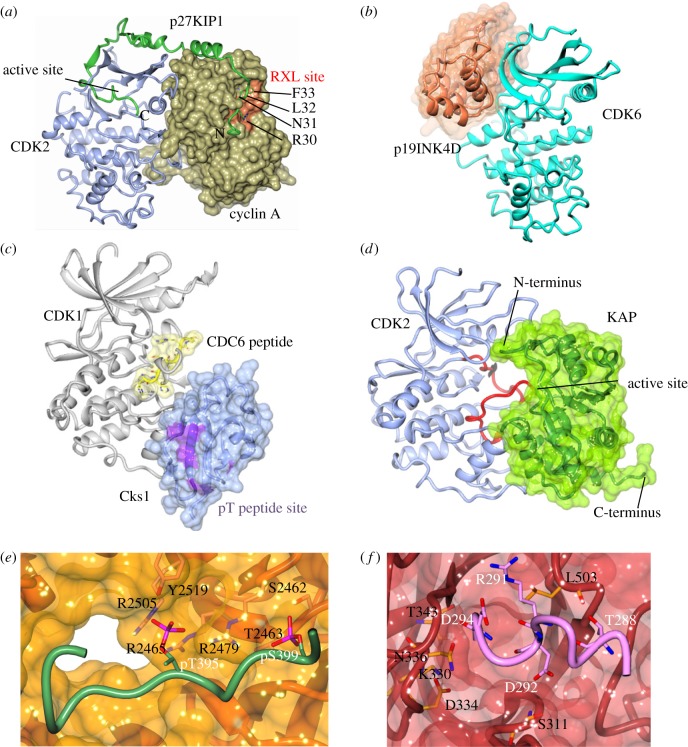
CDK–cyclin interaction partners. A number of CDK–cyclin partners and interaction sites have also been solved structurally. (*a*) CDK2–cyclin A p27KIP1 (PDB 1JST, CDK2–cyclin A, coloured as previous, p27^KIP1^ is coloured green and the hydrophobic patch of the RXL site is highlighted in orange with p27^KIP1^ side chains R30, N31, L32, F33 highlighted). (*b*) CDK6–p19INK4d (PDB 1BLX, CDK6, cyan; p19INK4D, orange). (*c*) CDK1–Cks1 (PDB 4YC6, CDK1, grey; CKS1, blue with phospho-threonine (pT)-interacting residues shown in purple; the peptide from 2CCI (yellow) has been superposed onto 4YC6). (*d*) CDK2-KAP (PDB 1FQ1, CDK2, blue with red activation loop; KAP, green). (*e*) cyclin E–Fbw7 (PDB 2OVQ, Fbw7, orange; cyclin E peptide, green). (*f*) cyclin D1–FBXO31 (PDB 5VZU, FBX031, crimson; cyclin D1 peptide, pink).

The ability of Cks1 to enhance the phosphorylation of a subset of CDK1 substrates was first recognized in *Xenopus* oocytes [130] and refined by further studies in *Saccharomyces cerevisiae* [131]. Cks1 binds to the CDK1 C-terminal lobe (figure 6*c*) and contains a phospho-threonine docking site that can recognize phosphorylated CDK1 substrates and promote their further hyperphosphorylation by CDK1 [132]. The order and pattern of target residue phosphorylation in multi-site phosphorylated substrates appears to be fine-tuned by the identity of the cyclin and the presence of Cks1 [131,133,134].

CDKs 7, 9, 12 and 13 phosphorylate the RNA polymerase CTD. The sequence of the CTD is unusual, being composed of 52 heptad repeats in humans, with the consensus sequence Y-S-P-T-S-P-S. Extracted from cells, CTD residues S2 and S5 are the most abundantly phosphorylated serine residues, while S7 is phosphorylated to a lesser extent [109,110]. The extent of phosphorylation within cells was found to be much less than expected, suggesting that multiple phosphorylation events within a single repeat or singly within adjacent repeats must be infrequent. Various studies have, together, suggested that the transcriptional CDKs have preferences for particular sites. For example, CDK7 has been shown to predominantly phosphorylate S5 and S7, CDK9 to have activity towards all three serines, and CDK12 and CDK13 to predominantly phosphorylate S2 [135]. Functionally significant interplay between phosphorylation sites has been shown for CDK9 where, using model three hepta-repeat substrates, S7 phosphorylation was found to prime subsequent CDK9-mediated phosphorylation. In this study, pre-phosphorylation of S2 or S5 blocked subsequent CDK9 activity and CDK9 preferentially phosphorylated S5 [108]. Unfortunately, there was no electron density to support binding of an S2 phosphorylated 13-mer substrate peptide following attempts to co-crystallize it with CDK13 [93]. To date, there is no detailed structural information to understand the molecular determinants that distinguish the activities of the CTD kinases towards their shared substrate and to what extent the complex local molecular environment impacts substrate selection.

Other CDK substrate docking sites have been identified but as yet structural information is lacking. Analysis of a set of *S. cerevisiae* Cln2 mutants has identified a surface shared with Ccn1 and Cln1 cyclin subtypes but not with Cln3 that recognizes a consensus substrate ‘LP motif’ that is enriched in leucine and proline residues [136]. Modelling the Cln2 structure on cyclin A reveals the docking site to be adjacent but non-overlapping with the RXL-binding site on the surface of the N-CBF. It is likely that ordered progression through the cell cycle results both from different CDK–cyclin pairings having different substrate selectivity and from the fact that the different CDK–cyclin pairings are expressed at different points in the cell cycle [137] (reviewed in [138]).

## 4. Regulatory protein interactions

### 4.1. Cell cycle CDK–cyclins: regulatory interactions determining activity

A number of cyclin-encoded protein-binding sites or short peptide motifs have been structurally characterized. A well-characterized example is the recycling of the cyclin RXL recruitment site that is exploited to either enhance or inhibit CDK activity. Alternatively, short motifs encoded within the cyclin sequence can be used both to dock cyclins to substrates to enhance CDK activity and alternatively to localize them to CDK regulators frequently resulting in a loss of CDK activity.

Members of the p27KIP1/p21CIP1 cyclin-dependent kinase inhibitor (CKI) family share an RXL motif with RXL-containing substrates and compete with them for CDK–cyclin association. The structure of a CDK2–cyclin A–p27KIP1 complex (PDB 1JSU, [117]) revealed the extended path of the N-terminal sequence of the intrinsically disordered p27KIP1 protein over the upper surface of the cyclin N-CBF (figure 6*a*). p27KIP1 then proceeds to disengage the edge β2-strand from the CDK2 N-terminal lobe and occupy the ATP-binding site, mimicking the interactions made by the adenine ring of ATP. p27KIP1 also acts as an assembly factor during G1 to assist the formation of active CDK4/6–cyclin D complexes, a role that also sequesters p21CIP1/p27KIP1 CKIs to promote G1 progression [27,139]. The retention of CDK activity in the presence of bound p27KIP1 is linked to the phosphorylation status of p27KIP1 Y88. Phosphorylation by tyrosine kinases (e.g. Src or Abl kinases) can generate CDK4/6–cyclin D–p27KIP1 [140–142] or CDK2–cyclin A–p27KIP1 [143] complexes that are catalytically active. The differences in kinetics and affinity of p27KIP1 and p21CIP1 binding to CDK2–cyclin A and to CDK4–cyclin D complexes may reflect an option for an alternative binding mode to CDK4 [144–146]. Exploiting NMR methods, p27KIP1 Y88 phosphorylation promotes the removal of the 3_10_ helix that occludes the CDK2 active site [143]. The structural basis of how phosphorylated p27KIP1 binds to CDK4/6–cyclin D to aid assembly of an active complex is yet to be elucidated by a co-complex structure.

The INK (inhibitors of CDK) family of CKIs selectively inhibits CDK4 or CDK6 and, through an allosteric mechanism, disfavours CDK–cyclin binding [15]. Their tandem ankyrin repeat structures exemplified by CDK6–p19INK4d ([147], PDB 1BLX; [148], PDB 1BI8) and CDK6–p16INK4a ([148], PDB 1BI7) bind in the vicinity of the CDK hinge on the interface opposite to the surface remodelled upon cyclin association (figure 6*b*). INK4 binding to CDK6 distorts the N-terminal kinase lobe relative to the C-lobe by approximately 15°, thus misaligning the key catalytic residues. The structures of individual INKs have also been determined by X-ray crystallography (p18INK4c, [149], PDB 1IHB) and (p19INK4d, [150], PDB 1BD8) or solution NMR (p15INK4b, [151], PDB 1D9S), (p16INK4a, [151], PDB 1DC2; p18INK4c, [152], PDB 1BU9; and p19INK4d, [153], PDB 1AP7).

The cell cycle CDKs are further distinguished by the CDK surfaces they exploit to regulate activity. For example, no protein equivalent to the INKs has been reported to bind to the CDK1/2 hinge. Similarly, there is no known protein that binds to CDK4 and CDK6 in a manner equivalent to the binding of Cks1 or Cks2 to CDK1 ([34], PDB 4YC6; figure 6*c*) or CDK2 ([154], PDB 1BUH). The CDK2 C-terminal lobe also recognizes kinase-associated phosphatase (KAP) that can dephosphorylate T160-phosphorylated CDK2 ([155], PDB 1FQ1; figure 6*d*).

In addition to helping to select mitotic substrate phosphorylation sites (see above), Cks1 collaborates with Skp2 to form the p27KIP1 phosphoT187-binding site within the SCF^Skp2^ (Skp1–cullin–F-box) E3 ubiquitin ligase complex ([156], PDB 2AST). This example is the first to show an F-box protein requirement for an accessory protein for substrate recognition [157,158]. Modelling studies using structures of sub-complexes show that a CDK2–cyclin A–p27KIP1–Cks1–Skp1–Skp2 complex can be built [156], but whether any subtle rearrangements occur will require determination of the structure of the CDK2–cyclin A–pT187p27KIP1–SCF^Skp2^ complex.

The LXCXE motif located towards the N-terminus of the D-type cyclins is highly conserved and represents an interesting example of a short cyclin-encoded motif that assists in substrate recruitment. D-type cyclins share this sequence with other cellular and viral proteins that bind to pRB [159]. In the CDK4–cyclin D1 structure, the motif is sequestered in the channel between the C-terminal CDK and cyclin lobes (figure 4*b*). However, the quality of the electron density map shows that it is flexible, suggesting it could disengage and remodel to bind to pRB. The structure of a complex of the pRB pocket domain and an LXCXE-containing peptide derived from the human papilloma virus E7 protein illustrates the interaction ([160], PDB code 1GUX). It is not known whether pRB and cyclin D engagement of LXCXE and RXL motifs, respectively, is synergistic or antagonistic for promoting pRB phosphorylation by CDK4 or CDK6, but it may be hypothesized to contribute to the mechanism that restricts CDK4/6 activity. Mutation of the LXCXE motif disrupts cyclin D1 activity in some cell line contexts where cyclin D expression has been reduced [161], but its mutation in a cyclin D1 ‘knock-in’ mouse study did not reveal any significant differences to the authentic cyclin D1 sequence [162].

### 4.2. Cyclin motifs regulating stability

Cyclin levels are tightly controlled and their degradation is a response to signalling pathway activation. Various E3 ubiquitin ligase complexes target cyclins for degradation, collectively employing short, flexible degron motifs to recognize their various cyclin substrates. The relationship between cyclin A- and B-containing CDK complexes and the anaphase-promoting complex/cyclosome (APC/C) illustrates this point [163]. Cyclins A and B are substrates of this E3 ubiquitin ligase and contain destruction (D) box (consensus motif RxxLx[D/E][Ø]xN[N/S], [164,165]) and KEN box (consensus motif [DNE]KENxxP) degron motifs [166], and in the cyclin A sequence, an ABBA motif (consensus motif KxxFxxYxDxxE, in cyclin A1 residues 132–143) mediates binding to the APC/C. The ABBA motif is also present in other proteins that bind to Cdc20 and Cdh1, both activators of the APC/C [167]. It has also been called a Phe box and was originally described in BubR1 [167–170].

Structural studies exploiting the fact that many APC/C inhibitors contain pseudo-substrate sequences that bind more tightly to the APC/C and its regulators than do its substrates have provided opportunities to visualize D-box, KEN box and ABBA motif binding to the APC/C. How D- and KEN-boxes bind to the Cdc20 β-propeller domain was revealed by the structure of the *Schizosaccharomyces pombe* mitotic checkpoint complex, the motifs being encoded in the BubR1/Mad3 subunit [171]. However, optimal D-box recognition requires an interface generated by an APC/C co-activator (Cdh1 or Cdc20) WD40 β-propeller domain and the APC/C subunit Apc10 [172]. The structure of a BubR1 KEN box-derived peptide bound to Cdc20 confirmed the nature of the KEN box–Cdc20 interface [173]. A complex of a peptide containing the ABBA motif (in this case derived from the *S. cerevisiae* APC/C inhibitor Acm1) provided a structural model for this cyclin A sequence, in this case binding to the alternative APC/C activator Cdh1 [174]. Blades 2 and 3 of the Cdh1 WD40 domain create a channel in which the peptide sits. As Acm1 also encodes a pseudo-substrate inhibitory KEN box motif, it also provided models for cyclin A and B engagement with Cdh1 through these sequences. The structure of the APC/C and its interactions with various of its regulators and substrates has been reviewed recently [175].

Members of an alternative family of E3 ubiquitin ligases, the Skp1–Cullin–F-box (SCF) complexes also recognize and degrade cyclins. Structures of cyclin E and cyclin D1 peptides bound to the F-box proteins Fbw7 and FBX031, respectively, reveal the diverse mechanisms employed. The cyclin E phospho-degron is encoded within the C-terminal tail (C-terminus at A410). Cyclin E is phosphorylated by glycogen synthase kinase 3 (GSK3) at T395 and undergoes autophosphorylation bound to CDK2 (at S399) to generate the phospho-degron motif recognized by Fbw7 [176,177]. A C-terminal 31 residue cyclin E phospho-peptide adopts an extended conformation straddling across the top of the WD40 propeller (figure 6*e*). Phosphorylated S399 and T395 are embedded in networks of hydrogen bonds, the phosphorylated S399 (S384 in paper) being more solvent accessible, whereas T395 (T380) is more buried within a shallow pocket.

Cyclin D1 phosphorylation at T286 by (*inter alia*) GSK3β [178] signals its degradation by promoting its nuclear extrusion (reviewed in [179]). However, cyclin D1 degradation is phosphorylation-independent when promoted through this genotoxic stress-induced pathway. Subsequent recognition of cyclin D1 by the E3 ubiquitin ligase SCF FBXO31 is not through direct binding of a phospho-T286-containing amino acid motif to FBXO31. Instead, the structure of the Skp1–FBXO31–cyclin D1 phospho-peptide (residues 279–295) complex revealed that essentially all the interactions between cyclin D1 and FBXO31 are made by the last four C-terminal cyclin D1 amino acids (292–295) and not the sequence immediately around T286 (figure 6*f*) [180].

### 4.3. Transcriptional CDKs: regulatory interactions exploiting alternative protein interaction sites

**Figure 7.**
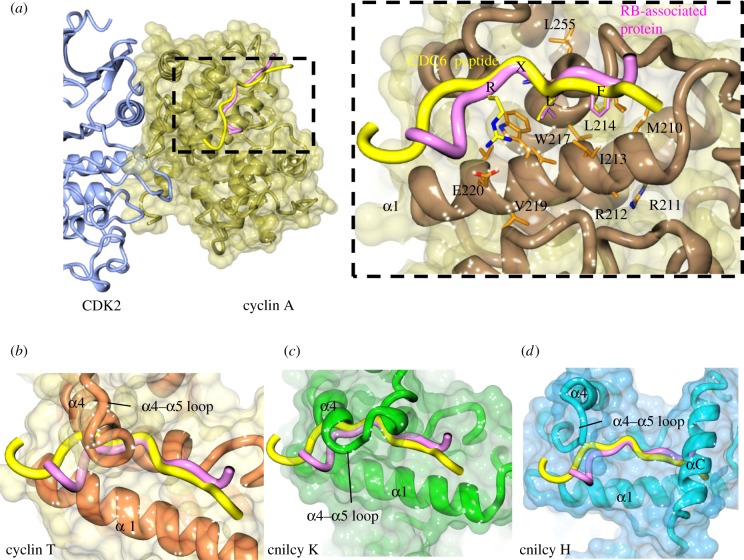
The RXL substrate recruitment site is unavailable in transcriptional cyclins. Differences between the cell cycle and transcriptional cyclin families within the N-terminal cyclin box fold (N-CBF) reveal different availabilities of the RXL site. (*a*) The cyclin A RXL site with CDC6 (yellow, PDB 2CCI) and RB-associated protein (pink, PDB 1H25) peptides bound is unavailable in transcriptional cyclins (*b*) cyclin T (PDB 3BLH, orange), (*c*) cyclin K (PDB 4UN0, green) and (*d*) cyclin H (PDB 1KXU, blue) due to extended α4 helices and protruding α4–α5 loops.

A comparison of the CDK–cyclin complexes regulating transcription illustrates ways in which the CDK–cyclin unit can be redeployed to expand the potential options for regulation by protein–protein interactions. The structural variety shown by P-TEFb transcription factor partners suggests that it may exploit multiple alternative interaction mechanisms. The determination of the monomeric cyclin T2 and CDK9–cyclin T1 structures revealed that the N-CBF recruitment site that is highly conserved in the cell cycle cyclins (A, B, D and E, figure 7*a*) is not present in cyclin T (figure 7*b*). The extra turn at the C-terminal end of cyclin T helix α4 folds over the surface of the N-CBF to occlude L43, the residue structurally equivalent to cyclin A W217, which forms the heart of the RXL-binding recruitment site. The loop linking helix α4 to α5 composed of residues H112–D123 is also extended when compared to the similar inter-helix sequence in cyclin A (T282–T287).

The absence of an N-CBF recruitment site is also a feature of the other cyclins that partner the transcriptional CDKs. Cyclin K shares extended α4 and α4–α5 loop structures with cyclin T, though the paths of the α4–α5 loops diverge ([181], PDB 2I53). But structurally, the effect is the same, and cyclin K F56 equivalent to cyclin A W217 is occluded from solvent (figure 7*c*). In the cyclin H structure ([182], PDB 1JKW and [183], PDB 1KXU), a shorter α4 helix and loop linking α4–α5 coupled with displacement of the N-terminal end of α5 relative to its position in cyclin T extensively remodel the cyclin H structure around R63, the residue equivalent to cyclin A W217 (figure 7*d*). However, the most significant difference imposed on the surface of the cyclin H N-CBF in this region is from the C-terminal helix that extends up from the C-terminal CBF (C-CBF) to make interactions with the loop linking the N-terminal helix and α1 of the N-CBF.

Taken together, these structural changes suggest that this set of cyclins must exploit alternative surfaces within their CBFs to mediate protein–protein interactions. That this is the case was first observed following the determination of the structure of CDK9–cyclin T in complex with HIV Tat. Tat promotes HIV transcription by competing with components of the inhibitory 7SK snRNP for P-TEFb association [184,185]. It recruits P-TEFb to the trans-activation response (TAR) element located at the 5′-end of the emerging HIV transcript, so that P-TEFb can phosphorylate and release the RNA Pol II for transcript synthesis [186–188].

**Figure 8.**
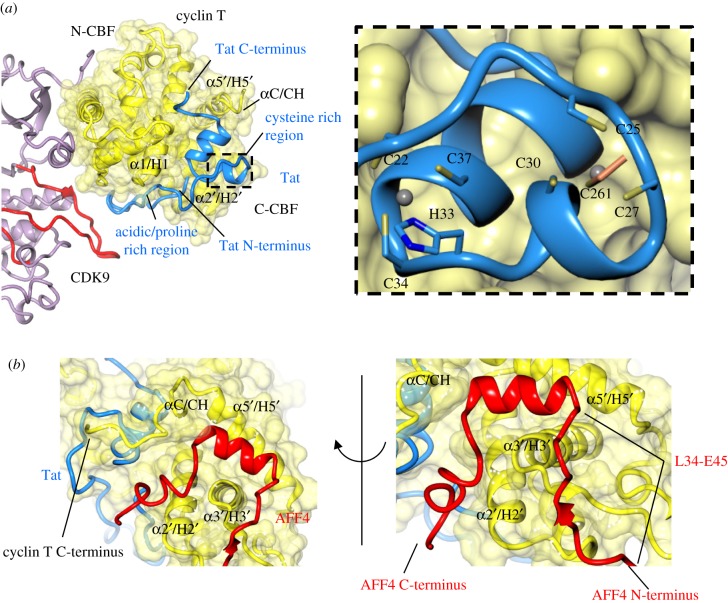
CDK9–cyclin T binds Tat and AFF4. (*a*) The HIV Tat protein binds to the C-terminal cyclin box fold (C-CBF) of cyclin T (PDB 3MI9, CDK9–cyclin T-coloured as previous; Tat, blue). Tat contains an acidic-/proline-rich region and a cysteine-rich region for the coordination of Zn, with the second site completed by cyclin T C261. (*b*) CDK9–cyclin T-Tat also binds AFF4 at the C-CBF (PDB 4OGR, AFF4, red).

Tat adopts an extended conformation and its structure is dictated by the multiple interactions it makes with P-TEFb generating a large buried surface area. It exploits the fact that CDK9 and cyclin T only interact through their respective N-terminal lobes to occupy the cleft they create between their C-terminal lobes and, in so doing, stabilizes the CDK9–cyclin T structure ([189], PDB 3MI9). The Tat acidic/proline-rich region binds within a depression between the two CBFs and then forms an extended open hairpin structure to head across to interact with the CDK9 activation loop. The cysteine-rich sequence and core are more compact and also bind into a groove between the CBFs. Two zinc ions are coordinated through multiple cysteine residues within the Tat sequence, the second zinc site completed by cyclin T1 C261 (figure 8*a*).

To what extent the viral protein is mimicking and exploiting authentic cyclin T interactions was appreciated with the determination of the structures of (i) CDK9–cyclin T–AF4/FMR2 Family member 4 (AFF4) ([190], PDB 4IMY), a scaffolding component of the super elongation complex (SEC) [191], (ii) CDK9–cyclin T–AFF4–Tat ([192], PDB 4OR5 and [190], PDB 4IMY) and ([193], PDB 4OGR) (figure 8*b*), and (iii) CDK9–cyclin T–AFF4–Tat–RNA (PDB 5L1Z). Tat binds to members of the SEC to rescue stalled RNA polymerase II during the transcription of the TAR element, and thus reinitiates the viral transcriptional regime [192]. AFF4 binds to cyclin T1 on the C-CBF, situated on the opposite side of cyclin T1 to the CDK9 interaction interface [190,192], although an individual AFF4 helix has been resolved behind the αD helix in the C-terminal lobe of CDK9 in several, but not all crystallographic copies.

AFF4 is an intrinsically disordered scaffolding protein that encodes short dispersed sequences that folds upon binding to dock to protein partners sequestering them together. The cyclin T-binding site is within the N-terminal 73 residues of AFF4 (figure 8*b*). From L34–E45, the AFF4 sequence extends along the lower edge of the cyclin T C-CBF, then folds to form a short helix that docks to make interactions along one helical face with cyclin T helix α5′ (C-CBF) and the C-terminal end of helix α3′ (C-CBF). Beyond L56, AFF4 nudges into the groove between the CBFs to contact Tat, the region being further shaped by a modification to the path taken by the cyclin T C-terminal sequence from that adopted in P-TEFb to accommodate the two proteins. Taken together, the interactions help to explain the observed enhanced affinity of Tat for P-TEFb bound to AFF4 than P-TEFb alone.

The binding of these two P-TEFb regulators to distinct but adjacent sites within the cyclin T C-CBF provides an opportunity for the integration of information from multiple signalling pathways that affect P-TEFb activity. Though structural details are lacking, it is known that the binding of hexamethylene bisacetamide (HMBA)-inducible protein 1 (HEXIM1), a component of the inhibitory 7SK snRNP particle [194], interferes with Tat binding ([195–197]), suggesting that its interaction is also mediated through the cyclin T C-CBF. The bromodomain protein 4 (Brd4) C-terminal P-TEFb-interacting domain (PID) has been reported to not only interact with cyclin T [198], but also both Brd4 [197] and HEXIM1 [199,200] have been proposed to also bind to CDK9, suggesting that the canyon between the two P-TEFb subunits might also be a hotspot for protein interaction.

### 4.4. Non-canonical cell cycle CDK–cyclin functions

#### 4.4.1. CDK4/6–cyclin D

In addition to their well-established cell cycle roles, CDK4, CDK6 and cyclin D also regulate many other aspects of cell behaviour such as transcription, cell metabolism [201–203], differentiation [204,205] and DNA repair (reviewed in [28,29,206,207]). Some of these functions are reported to require CDK4 or CDK6 kinase activity, but others apparently do not, suggesting that CDK4, CDK6 and cyclin D may, in certain contexts, act independently and scaffold or maintain the integrity of larger signalling complexes. Whether CDK4 and CDK6 can be cyclin D-associated but not have kinase activity remains to be determined [208,209]. By analogy with receptor tyrosine kinases, where downstream signalling is elicited by limited activity against a small set of spatially optimized substrates, it can be hypothesized that CDK4 and/or CDK6 roles in regulating transcription might result in some cases from their incorporation into large, chromatin-bound complexes at gene promoters where their substrates are co-located. The importance of these emerging CDK4/6 and cyclin D functions to disease is being revealed by proteomic analyses to characterize differences in CDK4/6 and cyclin D interactomes between normal and oncogenic states with the aim to identify changes promoting cell transformation (for example, see [77]).

Cyclin D isoform-specific functions distinguish the phenotypes of the cyclin D knockout mice [210] and are clearly important clinically (for example, see [211]). In some cases, these functions appear to be kinase independent. For example, D-type cyclins have been reported to act in a kinase-independent manner to antagonize the activity of the transcription factor DMP1 [212]. Subsequent studies have shown that D-type cyclins can enhance the transcriptional activity of, for example, the oestrogen receptor [213–215], but inhibit the activity of another hormone receptor, namely the androgen receptor [216–218]. Cyclin D can also engage with general transcription regulators and chromatin-modifying factors such as the histone acetyltransferase p300 [219] and can affect chromosome integrity [220]. The importance of cyclin D1 to the regulation of transcription has been highlighted in a recent proteomic study that identified cyclin D1-binding transcription factors in different organs during both normal mouse development and in tumorigenesis [221]. Cyclin D1 is also an important component of the cell's response to DNA damage, promoting repair [222–224]. Bound to chromatin, it can recruit RAD51 and localize to sites of DNA double-strand breaks through a BRCA2-dependent mechanism [225]. Tissue-specific roles of the D-type cyclins are also evident outside of cancer in the central nervous system [226–228], where cyclin D2, but not D1 or D3 knockout mice, are incapable of adult brain neurogenesis [226], suggesting a cell-cycle-independent role. Mutations to cyclin D2 T280 (equivalent to cyclin D1 T286) that prevent its phosphorylation by GSK3β and subsequent nuclear export leading to proteasomal degradation result in elevated cyclin D2 levels that cause megalencephaly–polymicrogyria–polydactyly–hydrocephalus syndrome (MPPH, a developmental brain disorder) [229]. Individuals with elevated cyclin D2 as a result of cyclin D2 mutation rather than activating mutations in the PI3 K–AKT–GSK3β pathway have an increased incidence of polydactyly, suggesting that characteristics of the cyclin D2 overexpression phenotype might also result from a potential role in regulating a programme of gene expression as well as promoting the aberrant expansion of neural precursors.

**Figure 9.**
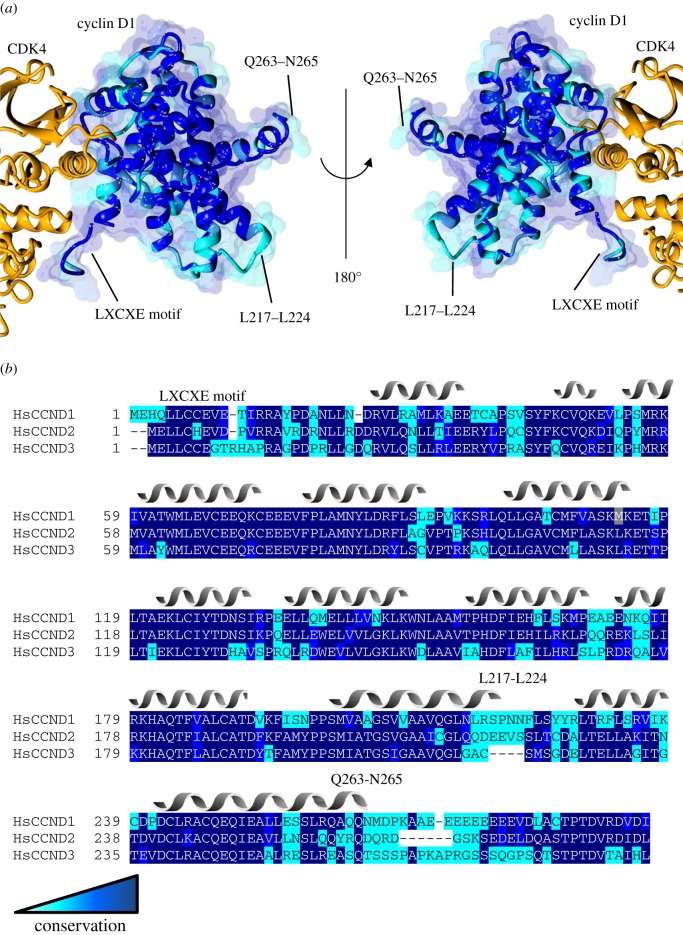
Cyclin D sequence conservation. (*a*) Sequence conservation between the cyclin D isoforms has been mapped onto the structure of CDK4–cyclin D1 (PDB 2W96) and is represented by blue-scale colouring. (*b*) Alignment of cyclin D1/2/3 conducted in Clustal Omega and output into ExPASy BoxShade. Secondary structure elements for cyclin D1 are shown above the sequence. CDK4 is coloured orange. The UniProt codes used for sequence alignment are: cyclin D1 (P24385), cyclin D2 (P30279) and cyclin D3 (P30281).

**Figure 10.**
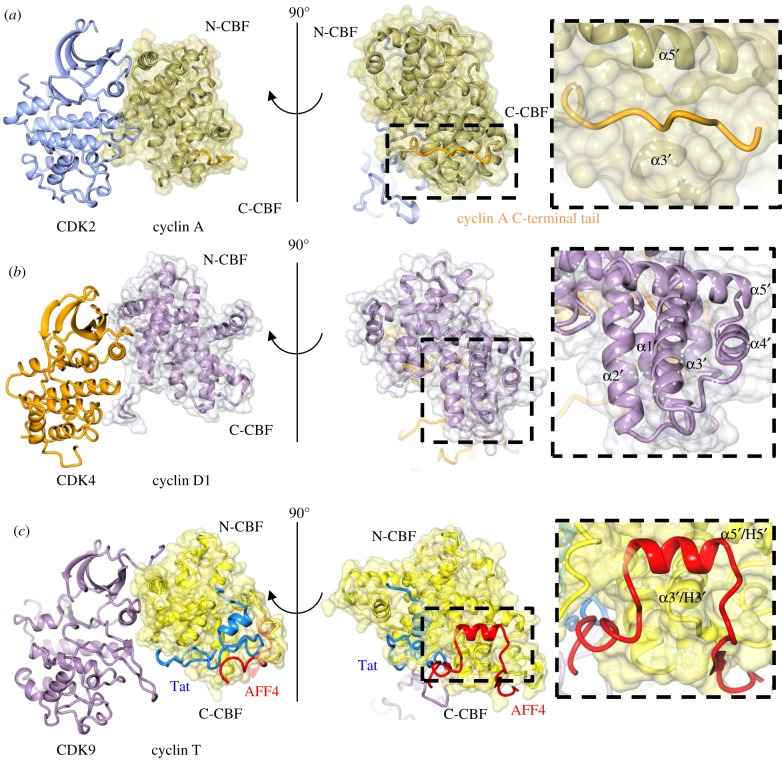
Comparison of cyclin T, cyclin D and cyclin A structures in the vicinity of the AFF4-binding site. (*a*) CDK2–cyclin A showing the C-CBF with the C-terminal cyclin A tail (orange) accommodated (PDB 1FIN, CDK2–cyclin A coloured as previous). The same site is presented for (*b*) cyclin D1 (PDB 2W96, coloured as previous) and (*c*) cyclin T (PDB 4OGR, coloured as previous), which is known to accommodate the binding of both AFF4 and Tat.

A comparison of the structures of cyclin D1 and cyclin D3 illustrates the extent of sequence conservation between the three isoforms and reveals the locations of conserved isoform-specific surfaces (figure 9). Many of the transcription factors that bind to cyclin D do not encode an obvious RXL motif, suggesting that they may employ an alternative binding site on the cyclin D surface. It can also be hypothesized that some of these interactions may be indirect, for example bridged through binding of cyclin D to RXL-containing transcriptional regulators such as members of the E2F family. Many activities of cyclin D in transcription are reported to be kinase-independent, which suggests that the cyclin D CDK4/6 interface may be accessible, although a kinase activity-independent scaffolding role of a CDK–cyclin D complex cannot be excluded. Given the structural similarities between the relative dispositions of the CDK and cyclin subunits in CDK4–cyclin D1/D3 and CDK9–cyclin T (figure 10), it remains a possibility that a protein interaction site on the cyclin C-terminal lobe is conserved between cyclin T and cyclin D.

#### 4.4.2. Cyclins E and A

Both cyclin A and cyclin E have also been reported to have CDK-independent roles suggesting scaffolding or regulatory functions. However, structural details as to whether these protein interactions require the RXL-binding site on the N-CBF overlap with other parts of the p27KIP1-interacting surface or employ novel binding sites are not known. Similarly, there are structurally unverified reports of cell cycle regulators binding to RNA. As examples, CDK2 and p21CIP1 have been reported to bind to Foxo3 circular RNA [230] and cyclin A2 to the 3′-untranslated region (UTR) of Mre11 mRNA [231]. This latter interaction is independent of an associated CDK partner and regulates Mre11 translation. Mutational analysis mapped the binding site to the C-CBF at a surface not previously implicated in protein association, suggesting that the surface of cyclin A may be more widely exploited than previously thought.

A number of studies have highlighted potential kinase-independent functions of cyclin E [232–234]. Cyclin E1 and E2 knockout mice are, respectively, viable or infertile in males, and double knockouts are embryonic lethal [232,235]. These phenotypes demonstrate the necessity for at least one E-type cyclin in the embryo. Mutations to alanine within the CDK2-binding interface of cyclin E, in a loop region between helices H3 and H4, permit weak, p21Cip1/p27Kip1-dependent binding to CDK2, but abolish cellular kinase activity. These kinase-activity-deficient mutants re-established the observed transformative potential of cyclin E and restored MCM protein loading onto the pre-replication complex to facilitate G_0_–S-phase transition [232]. Cyclin E also localizes to centrosomes independently of CDK2 [236], which may be relevant to centrosome duplication [237]. In terms of cancer transformative potential, analysis in rat embryonic fibroblasts has also suggested that this property of cyclin E may, in certain circumstances, be independent of CDK2 [234], an observation that is also consistent with analyses conducted in hepatocellular carcinoma (HCC) [233]. Cyclin E1^−/−^/E2^−/−^ mice stopped tumour cell proliferation in clonogenic assays [233], while the individual function of cyclin E subtypes was resolved in hepatocyte-specific NEMO and global *CCNE1* or *CCNE2* knockout mice [238]. Cyclin E1, and not cyclin E2, was shown to be coupled with liver disease and hepatocarcinogenesis in this model system [238]. The kinase-independent nature of cyclin E in HCC progression was also highlighted by the finding that CRISPR/Cas9 CDK2 deletion and kinase dead forms of CDK2 were not sufficient to abolish cell growth [233]. These data appear to contrast with evidence from cyclin E amplified high-grade serous ovarian carcinoma, which suggest that these particular subtypes are sensitive to CDK2 knockdown through RNA interference [239,240]. Taken together, these results suggest that cyclin E has kinase-independent roles and that there are subtle differences by which cyclin E and its CDK–partner CDK2 are exploited in cancer progression. Again, whether uncharacterized CDK2– and cyclin A or E–protein interaction sites mediate these activities awaits further study.

## 5. Aberrant mutations/processing—structures relate to dysregulated function

CDK–cyclin-containing protein complexes have been implicated in a range of disease settings [8,241,242]. In cancer, in particular, therapeutic design and development has been directed at targeting members and regulators of the cell cycle CDK–cyclin families [25,30,72,243], with emphasis on combatting phenotypes driven by genetic amplification of CDK or cyclin family proteins or genetic deletion of their regulators (e.g. the INK4 family for CDK4/6 [27]). In addition to genetic amplification, structural alterations through point mutation are also evident and may be relevant to the subcellular function of these enzymes within the cancer microenvironment.

**Figure 11.**
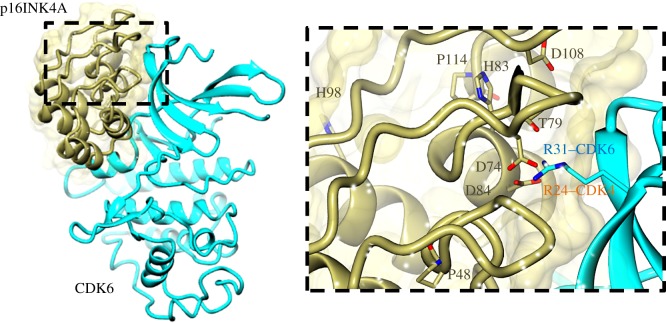
Point mutations in CDK4/6–p16INK4A. CDK4/6 and p16INK4A (represented here by CDK6, PDB 1BI7, CDK6, cyan; p16INK4A, gold) contain a number of residues that are frequently mutated in cancer. Several commonly described mutations occur on the CDK–p16INK4A interface, disrupting the complex and leading to kinase dysregulation. Other p16INK4A residues such as H98 and P48 are located further from the CDK-binding interface. CDK6 mutations are selected from those described in cancer genome repositories (see the text for further details).

Mutation of CDK4 R24 to C/H/L/S, first described in melanoma [244–246], and documented in a further 27 samples in the cBioPortal database [247,248], is known to increase kinase activity (reviewed in [249]). R24 is located on β2 of the N-terminal lobe of CDK4 and abolishes binding to p16^INK4A^ [139,250]. The corresponding arginine in CDK6, R31, coordinates through hydrogen bonds to several p16INK4a polar/acidic residue side chains, namely D74, T79 and D84, which may, in turn, be stabilized by R87 of p16INK4A ([148], PDB 1BI7) (figure 11). As the sequences of CDKs 4 and 6 are highly conserved within the N-terminal lobe, it is anticipated that mutation of CDK4 R24 also abolishes p16INK4A association by breaking these key interactions, although this hypothesis is yet to be confirmed by determination of a CDK4–p16INK4A structure. That this interaction is vital to CDK4/6–p16INK4a association is confirmed by reciprocal mutations to D84 in p16INK4a, one of several proposed mutational hotspots [251]. Mutation drives aberrant activation of CDK4/6–cyclin D [252]. The p16INK4a D84N mutant shows a stark increase in CDK4 activity relative to WTp16INK4a in an Rb phosphorylation assay [253], and limited ability to bind to CDKs 4 and 6 in cell-free biochemical direct binding analyses [78].

Consultation of cancer genome repositories such as cBioPortal [247,248], the COSMIC database [254] and TumorPortal [255] reveals a variety of other missense mutations within CDK–cyclins in the context of cancer (e.g. R168C in CDK5, R86Q CDK9, R378G in cyclin A2). However, a number of these are insufficiently characterized (mutations reviewed in [256]). In a number of cases, this results from the mutations being located outside of structured or crystallographically resolved regions, making constructs difficult to interrogate biophysically/biochemically, while in other instances it is amplification/upregulation of the CDK–cyclin component rather than mutation that is likely to drive proliferation.

Aside from mutations to key binding-partner interaction sites, other aberrant processing of transcripts can also lead to impaired cellular function of CDK–cyclins. In several tumour types, the A/G870 polymorphism within the *CCND1* transcript can result in alternate splicing [257]. A/G870 is located at the end of exon 4 before intron 4 within the 5-exon long *CCND1* DNA sequence. The A870 polymorphism is reportedly more likely to lead to an alternative *CCND1* transcript, which in turn codes for the translation of cyclin D1b protein [258,259]. Cyclin D1b includes additional residues encoded by intron 4 and thus is bereft of key regulatory residues at the C-terminal end [257,260]. These residues include the PEST motif (named using the single letter amino acid code) and T286, important for protein degradation and nuclear export, respectively [260], as well as the LXXLL motif that is involved in cyclin D transcriptional function [257]. As noted above, this region is flexible or unstructured and not visible in the CDK4–cyclin D3 electron density map. Mutations to equivalent residues in cyclin D2 (T280) and cyclin D3 (T283) have also been reported for a small subset of acute myeloid leukaemia [261] and Burkitt lymphoma [262] sufferers, respectively. Indeed, these mutated proteins present with similar phenotypes to cyclin D1b expressing cells, showing adverse degradation and enhanced nuclear localization [261–264].

In addition to DNA mutations in CDK–cyclin partners, aberrant post-translational processing is strongly linked to dysregulated function. One particular example in the context of cancer are the low-molecular-weight forms of cyclin E1, though interestingly not cyclin E2 [265,266]. Cleaved post-translationally by elastase [267], low-molecular-weight forms of cyclin E1 facilitate increased kinase activity, potentially through increased CDK2 affinity [267,268]. The low-molecular-weight forms are cleaved within the sequence N-terminal to the known structured CBFs, and thus, any structural rationale for differences in affinity for CDK2 between full-length and low-molecular-weight forms remains to be elucidated. Whether cyclin E1 also contains additional N-terminal regulatory motifs, reminiscent of those seen in cyclins A and B such as the ABBA [167] or D-box [269] motifs, and whether these sequences are lost in low-molecular-weight forms remain to be confirmed.

## 6. Macromolecular CDK-containing complexes and electron microscopy: the future

While an enormous wealth of detail has been revealed by X-ray crystallography studies, the question of how CDK–cyclin partners participate in larger macromolecular complexes is yet to be fully answered. However, cryo-electron microscopy (cryo-EM) is emerging as a technique that can address this deficit, and several CDK–cyclin-containing complexes have been determined.

Transcription factor IIH (TFIIH) is a large 10 subunit complex recruited by RNA polymerase II (RNA pol II) during transcription initiation and is also important in nucleotide excision repair (NER) [6,270]. The CAK complex of CDK7–cyclin H and Mat1 is known to be required for phosphorylation of RNA pol II CTD, but is removed from TFIIH during NER [271,272]. However, the binding of CDK7–cyclin H to Mat1 is not fully resolved within the TFIIH structure [273], which may reflect the ability of CAK to disengage from TFIIH. The extended helical structure of Mat1 links the TFIIH ATPase and helicase subunits XPD and XPB [273].

TFIIH is also regulated by another CDK-containing complex, termed the Mediator complex [274]. The Mediator complex contains approximately 30 polypeptide chains and has a molecular weight of greater than 1 MDa. It is formed from four distinct modules: the head, middle and tail modules, and the reversibly bound CDK8 kinase module (CKM), which can contain CDK8 or CDK19 bound to cyclin C [275–277]. CDK8–cyclin C inhibits RNA pol II CTD phosphorylation by TFIIH through phosphorylation of cyclin H at the extreme N- and C-terminal helices (on residues S5 and S304, respectively) [274]. Using EM, the structure of the yeast CKM revealed that cyclin C, and not CDK8, binds to subunit MED12. The importance of this interaction is highlighted by mutations to the cyclin C–MED12-binding interface that inhibit Mediator activity and have been linked to uterine leiomyoma [278]. MED12 bridges CDK8–cyclin C with MED13, which in turn associates with MED19 of the middle module. CDK8 is shown to associate with the head module around subunits MED18–20 [279]. The central modules of Mediator have been revealed recently in a cryo-EM determined structure at 4.4 Å resolution [276,280].

**Figure 12.**
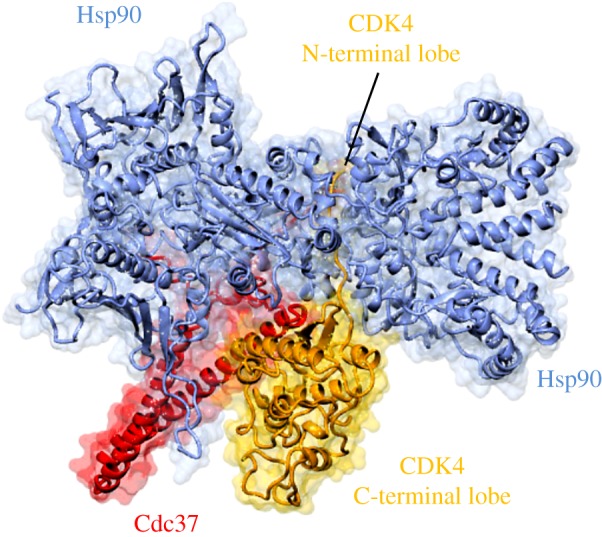
The cryo-EM structure of the CDK4–Cdc37–Hsp90 complex. Both copies of Hsp90 are coloured blue; Cdc37 is drawn in red and CDK4 in orange. Structure is drawn from PDB 5FWK.

Inactive kinase conformations within larger complexes can also regulate function. An example of this phenomenon is offered by CDK4 and the Hsp90 chaperone system [79]. Hsp90 protein kinase clients are selectively recruited through the co-chaperone Cdc37 to form a complex that holds the kinase in a protected state until it can be relinquished to an appropriate partner [281–283]. Indeed, in the cryo-EM structure of the Hsp90–Cdc37–CDK4 complex (figure 12), CDK4 is partially unfolded at the N-terminus, where β5 of CDK4 has lost secondary structure and winds into Hsp90 [79]. Cdc37 also supports CDK4 at the C-terminal lobe between the αC-helix and the loop linking to β4. CDK4 is a stronger Cdc37–Hsp90 client than CDK6 [76], and this is reflected in the ease with which the CDK can be handed off to partner proteins such as the D-type cyclins and members of the INK4 family [78].

## 7. Concluding remarks

The expansion of the CDK family from a single essential CDK in lower eukaryotes has enabled individual CDKs to develop tissue-specific functions and to respond more sensitively and selectively to intra- and intercellular signals. Structural studies have revealed their distinguishing features and help to provide explanations for their mechanistic differences. CDK–cyclin complexes have proved to be more diverse than was originally envisaged. This structural diversity has recently been successfully exploited to identify the first CDK inhibitors to be registered for clinical use targeting CDK4 and CDK6 (reviewed in [32,72]). ATP-competitive CDK inhibitors that selectively target other family members similarly exploit sequence differences within the active site and/or unique conformations that permit optimization of inhibitor–CDK interactions that discriminate the family members. Whether these inhibitors will be useful in the clinic will require careful target validation studies to identify cellular settings in which aberrant CDK activity is the cancer driver.

For the future, more specific chemical probes and selective antibodies are now required to provide greater understanding of CDK roles outside of the cell cycle, in particular understanding the links between their roles controlling the cell cycle and cell differentiation. Another exciting development is the application of electron microscopy to study larger CDK-containing complexes. These structures will further our understanding of CDK regulation and may well provide additional opportunities to more selectively inhibit CDK activity in clinically relevant settings.

## Data accessibility

This article has no additional data.
